# Digit Amputation Secondary to COVID-19-Induced Raynaud’s Phenomenon

**DOI:** 10.7759/cureus.76204

**Published:** 2024-12-22

**Authors:** Rui Carvalho, Joana Subtil, Elisa Macedo Brás, Jacinta Campos, Renata Silva

**Affiliations:** 1 Internal Medicine, Centro Hospitalar de Trás-os-Montes e Alto Douro, Vila Real, PRT; 2 Vascular Surgery, Centro Hospitalar de Trás-os-Montes e Alto Douro, Vila Real, PRT

**Keywords:** amputation, digit necrosis, infection triggered vascular phenomenon, post-covid-19 sequelae, raynaud’s phenomenon

## Abstract

Raynaud’s phenomenon (RP) is characterized by episodic vasospasm of the small blood vessels, primarily affecting the fingers and toes. Management includes lifestyle modifications, pharmacological treatments, and in severe cases, surgical interventions.

Here we report a case of an 80-year-old male patient with a history of hypertension, dyslipidemia, obesity, and atrial fibrillation who presented to the emergency department with edema, cyanosis, and intense pain in the fingers of both hands following a mild COVID-19 infection (no dyspnea or hypoxemia). Despite stable vital signs and an otherwise unremarkable physical exam, laboratory tests revealed leukocytosis, elevated inflammatory markers, and signs of local infection. Consequently, he was admitted to the ward on antibiotics (amoxicillin/clavulanic acid), nifedipine for vasodilation, and opioids for pain control. However, his condition deteriorated, with progressive necrosis necessitating escalation of therapy to iloprost infusion and bosentan. The right hand showed no improvement, and surgical intervention was planned around wound care aimed at mummification and subsequent amputation. The left hand gradually improved with continued medication. During hospitalization, serologic testing and nailfold capillaroscopy did not reveal any secondary causes such as connective tissue diseases. Imaging studies ruled out underlying neoplasia and thrombosis. The patient was diagnosed with severe Raynaud’s phenomenon, potentially triggered by the recent COVID-19 infection, a phenomenon already described in case reports.

This case underscores the potential impact of COVID-19 as a precipitating factor for severe Raynaud’s phenomenon, particularly in elderly patients with comorbidities. The rapid progression and refractory nature of the condition highlight the need for early recognition and aggressive management.

## Introduction

Raynaud’s phenomenon is a condition characterized by episodic vasospasm of the small blood vessels, primarily affecting the fingers and toes, in response to cold or emotional stress. This vasospasm leads to a sequence of color changes in the skin: white (pallor) due to lack of blood flow, blue (cyanosis) from prolonged lack of oxygen, and red (rubor) upon reperfusion [1-2].

The prevalence of Raynaud’s phenomenon in the general population has been reported to be approximately 5% [3].

Raynaud’s phenomenon can occur either as a primary (idiopathic) phenomenon or secondary to a wide range of underlying medical conditions, most commonly connective tissue diseases. Secondary Raynaud’s phenomenon is often associated with more severe outcomes, including digital ulceration and ischemia [3,4]. The connection between COVID-19 and Raynaud's phenomenon has been noted in various situations, such as direct infection with SARS-CoV-2 and post-vaccination events. However, the exact pathophysiological mechanisms remain unclear, requiring further investigation and ongoing monitoring [5]. Case reports have indicated that COVID-19 infection may contribute to the onset of Raynaud’s phenomenon. One report discussed a worker exposed to vibration who developed Raynaud’s phenomenon after a mild COVID-19 infection [5]. Another case report highlighted the progression from Raynaud’s phenomenon to systemic sclerosis following SARS-CoV-2 infection, suggesting that viral infections, including COVID-19, might act as triggering factors for autoimmune [6].

Educating patients and encouraging lifestyle changes are the primary approaches to treating Raynaud's phenomenon. A variety of pharmacological treatments, both oral and intravenous, are available for managing this condition. First-line pharmacologic treatment typically includes calcium channel blockers, such as nifedipine, which have been shown to significantly reduce the frequency and severity of Raynaud attacks [7]. Second-line pharmacologic treatments include phosphodiesterase 5 inhibitors (such as sildenafil), endothelin receptor antagonists (such as bosentan), and prostacyclin analogs (such as iloprost). These medications are especially beneficial for patients with critical ischemia or those who have not responded adequately to calcium channel blockers [7].

In cases where Raynaud's phenomenon is resistant to treatment and leads to tissue ischemia, surgical intervention may be necessary [8].

## Case presentation

An 80-year-old male patient, with a history of hypertension, dyslipidemia, obesity, atrial fibrillation (AF), hyperuricemia, benign prostatic hyperplasia, and prediabetes was being treated with atorvastatin 20 mg once daily, metoprolol 100 mg once daily, apixaban 5 mg twice daily, perindopril 10 mg once daily, chlortalidone 50 mg once daily, amlodipine 5 mg once daily, pantoprazole 40 mg once daily, dutasteride + tamsulosin 0.5 mg + 0.4 mg once daily, and allopurinol 300 mg once daily.

He presented to the emergency department on January 3rd, due to edema of the fingers of the right hand associated with cyanosis of the fingertips and intense pain after an initial period of pallor, followed by the appearance of darkened areas and progression of the same symptoms to the left hand. He had a history of COVID-19 infection with mild symptoms, two weeks prior, and no history of trauma, wounds, or of prior Raynaud's phenomena.

On physical examination, vital signs were stable: blood pressure 118/80 mmHg, heart rate 92/min, SpO2 (room air) 96%, temperature of 36.8°C. The patient was alert, oriented, and cooperative. The chest was symmetric with no respiratory effort. Breath sounds were clear to auscultation bilaterally. Heart sounds were regular with normal S1 and S2 and no murmurs. Lower limbs were with edema up to the knee. Both hands appeared with cyanotic distal extremities, cold, swollen, and painful; only the first finger was spared. There was no tenderness or swelling of the finger joints.

Initial laboratory tests were mostly unremarkable apart from a slightly elevated C-reactive protein. The results are shown in Table 1.

**Table 1:** Initial laboratory workup of the patient.

Test	Result	Reference range
Haemoglobin (Hb)	14.5 g/dL	12-16 g/dL
Leucocyte count	13.860 /uL	4000-11 000/uL
Neutrophils	79.1%	53.8-69.8%
Platelets count	280.000 /uL	150 000-400 000/uL
Urea	68 mg/dL	<50 mg/dL
Creatinine	1.30 mg/dL	0.6-1.1 mg/dL
Aspartate aminotransferase (AST)	53 U/L	<35 U/L
Alanine aminotransferase (ALT)	34 U/L	<33 U/L
Gamma-glutamyl transferase (GGT)	59 U/L	7-32 U/L
Alkaline phosphatase (ALP)	135 U/L	35-105 U/L
Lactate dehydrogenase (LDH)	515 U/L	135-214 U/L
Total bilirubin	0.40 mg/dL	<1.2 mg/dL
C-reactive protein (CRP)	9.70 mg/dL	<0.5 mg/dL
D-dimers	1.42 ug/mL	<0.5 ug/mL
Uric acid	5.2 mg/dL	3.6-7.0 mg/dL
N-terminal pro b-type natriuretic peptide (NT-proBNP)	2199 ug/ml	<120 ug/ml
Fasting blood glucose	103 mg/dL	75-100 mg/dL
Glycosylated hemoglobin (HbA1C)	6.2%	<6%
Fibrinogen	795 mg/dL	200-400 mg/dL
Activated partial thromboplastin time (aPTT)	37.3 seconds	25-35 seconds
International normalized ratio (INR)	1.03	<1.2

**Figure 1:**
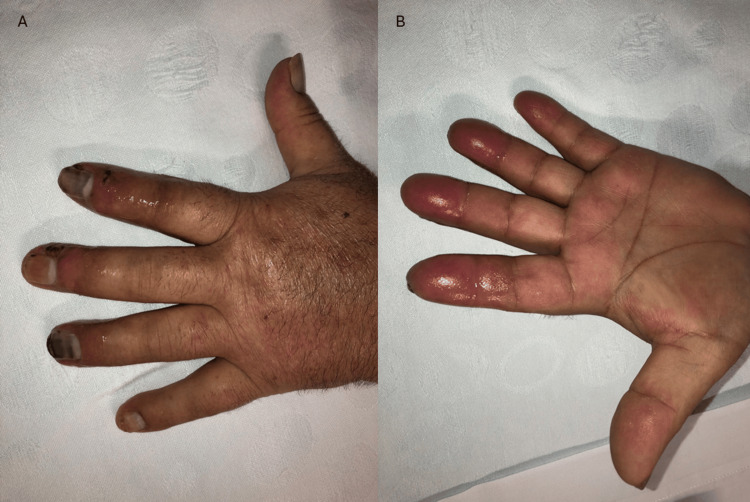
(A) Dorsal surface of the left hand, showing trophic lesions with exudate discharge, joint edema, cyanosis and erysipela. (B) Palmar surface of left hand, showing edema and erysipela.

The patient was evaluated by general surgeons, presenting with patent symmetrical radial and ulnar pulse. There were trophic lesions with exudate discharge, joint edema, cyanosis, and dermohypodermitis from the second to the fourth finger, in both hands. The left hand of the patient at the initial presentation can be seen in Figure 1.

The patient was admitted to the hospital for severe Raynaud's phenomenon in both hands with digital ulcers and signs of infection. It was his first episode, with a symmetrical involvement of both hands, saving his thumb. Blood cultures were collected and empirical antibiotic therapy with amoxicillin/clavulanic acid 1.2 g 8/8 h was started. Also, nifedipine 30 mg twice daily was started with opioids for pain control.

**Figure 2:**
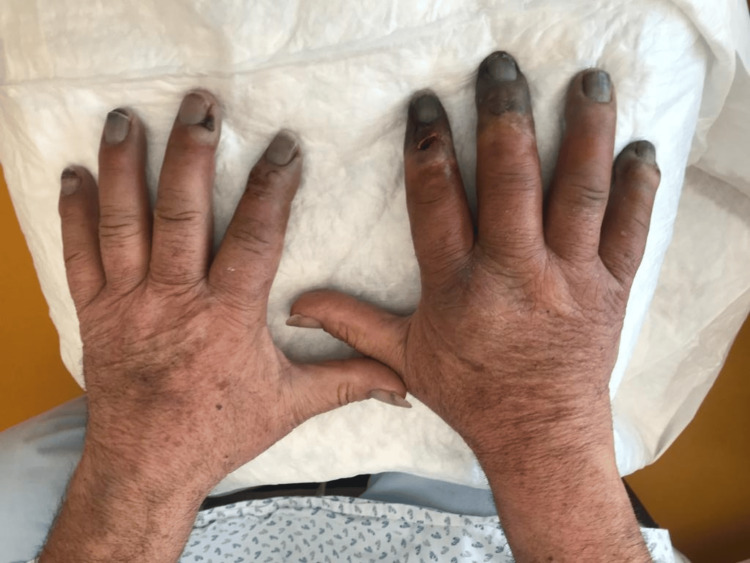
Dorsal surface of both hands following initial treatment, showing on the right signs of necrosis of 2nd-5th fingers.

Despite the instituted treatment, the patient continued to experience intense pain without pathogen identification, with progressing cyanosed extremities and subsequent necrosis as we can see in Figure 2 (one week after initial admission), therefore it was decided to start iloprost infusion for five days and bosentan.

During hospitalization, serologic testing was performed (Table 2).

**Table 2:** Serologic workup of the patient.

Test	Result	Reference range
Erythrocyte sedimentation rate (ESR)	56	<40 mm/1ªh (age-adjusted)
Antinuclear antibody (ANA) screen	< 1:160	< 1:160
Rheumatoid factor screen	7	<14 U/mL
Complement C3c	174	90-180 mg/dL
Complement C4	38	12-36 mg/dL
Beta-2 glycoprotein IgG	4.5	<10 U/mL
Beta-2 glycoprotein IgM	< .9>	<10 U/mL
Anti - cardiolipin IgG	1.2	<40 GPL/mL
Anti - cardiolipin IgM	3.4	<40 MPL/mL
Lupus anticoagulant	Positive; after 12 weeks negative	
Anti-cyclic citrullinated peptide (anti-CCP)	1.1	<7 U/mL
Anti - Scl 70 (anti-topoisomerase I)	Negative	
Anti - CENP A (circulating anticentromere A)	Negative	
Anti - CENP B (circulating anticentromere B)	Negative	
Anti - RP 11 (RNA polymerase III)	Negative	
Anti - RP 155 (RNA polymerase III)	Negative	
Anti – fibrillarin	Negative	
Anti - NOR 90 (Nucleolar Organizer Region 90)	Negative	
Anti - Th/To (Th/To ribonucleoprotein)	Negative	
Anti - PM - Scl 100 (polymyositis and scleroderma)	Negative	
Anti - PM - Scl 75 (polymyositis and scleroderma)	Negative	
Anti – Ku (Ku DNA-binding protein)	Negative	
Anti – PDGFR (platelet-derived growth factor receptor)	Negative	
Anti - Ro 52	Negative	
IgG	1544	600-1560 mg/dL
IgM	36	30-360 mg/dL
IgA	103	90-410 mg/dL
Cryoglobulins	Negative	
Human immunodeficiency virus (HIV)	Negative	
Hepatitis B virus (HBV)	Negative	
Hepatitis C virus (HCV)	Negative	
Blood cultures	Negative	

Nailfold capillaroscopy was also performed; however, due to extracellular matrix edema and necrosis, there was poor visualization of the capillaries. Nevertheless, there do not appear to be megacapillaries or microhemorrhages.

After finishing the iloprost infusion (for five days, titrated to a maximum of 160 ng per minute), treatment with bosentan 125 mg once daily and nifedipine 30 mg once daily was continued.

**Figure 3:**
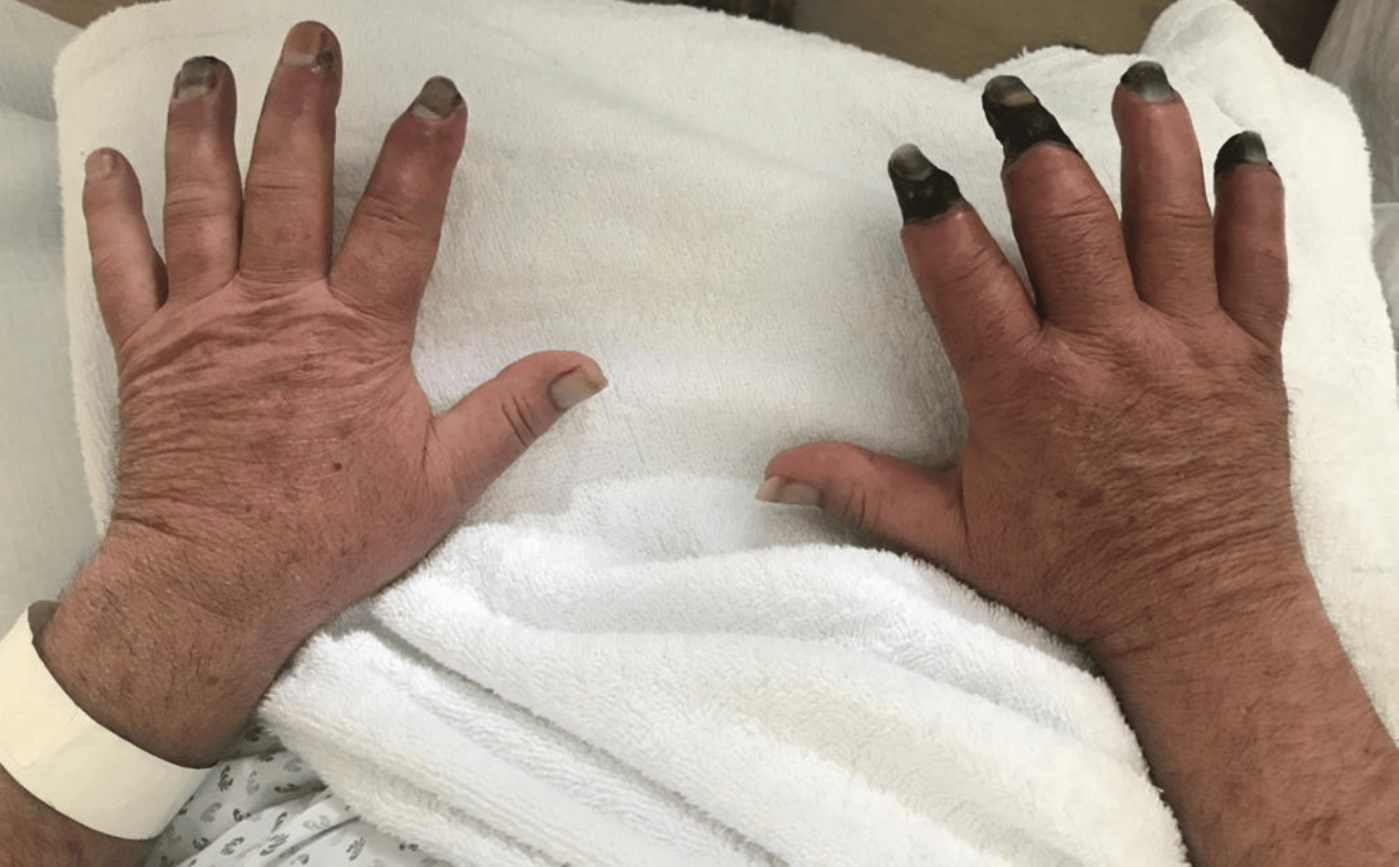
Dorsal surface of both hands; left hand with resolution of exudative lesions, right hand showing dry necrosis of 2nd, 3rd and 5th fingers.

The right hand did not show clinical improvement, maintaining progressive ischemia. The patient was evaluated by Vascular Surgery, with the decision to proceed with wound care aimed at mummification and subsequent amputation. The left hand showed gradual clinical improvement, with exudative lesions in resolution. The hands are shown in Figure 3.

**Figure 4:**
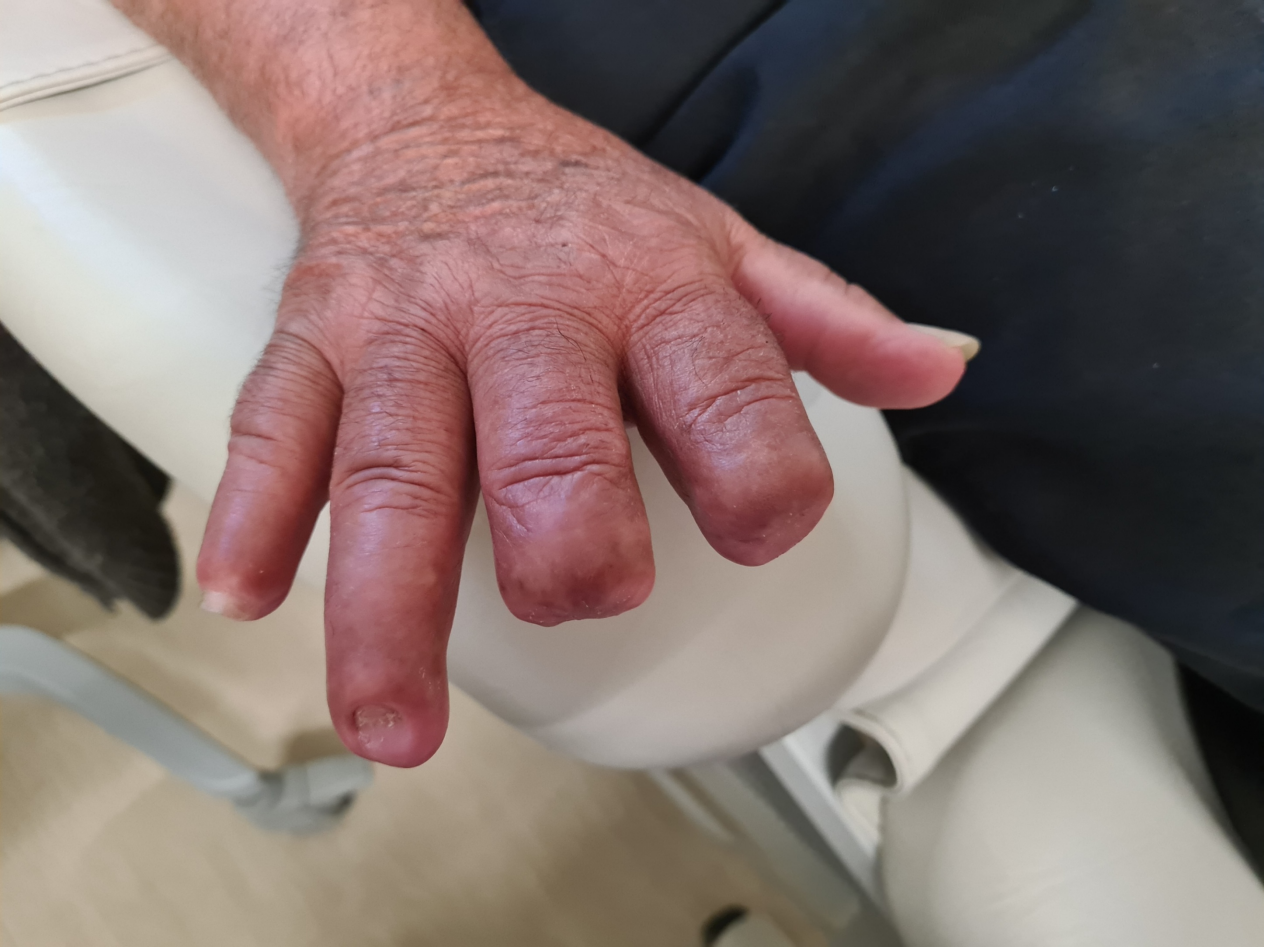
Dorsal surface of right hand after amputation of 2nd, 3rd and 5th fingers.

At the time of discharge, the patient maintained treatment with bosentan and nifedipine and wound care. He continued follow-up with physicians and vascular surgeons and was submitted to amputation of the necrotic fingers six months after diagnosis. The final result can be seen in Figure 4.

## Discussion

This case presents a complex and severe manifestation of Raynaud's phenomenon in an 80-year-old male patient. The patient’s first episode of Raynaud's phenomenon occurred two weeks following a mild COVID-19 infection without other apparent etiology, suggesting a potential link between the viral infection and the onset of the vascular condition.

Initial management included empirical antibiotic therapy with amoxicillin/clavulanic acid, given the signs of infection, and nifedipine for vasodilation. Despite this, the patient’s condition deteriorated, leading to the initiation of iloprost infusion and bosentan. These advanced therapies aimed to address the severe ischemia and potential underlying endothelial dysfunction, however, the right hand showed no improvement and continued to progress to necrosis, necessitating a surgical consultation. After the infection was resolved, the decision to pursue wound care aimed at mummification prior to amputation reflects a pragmatic approach to managing the extensive tissue damage. In dry gangrene, autoamputation is often the preferred option, particularly for patients who are not good candidates for surgery, especially high-risk patients such as diabetics, and the elderly [9]. This strategy aims to control infection, limit further tissue loss, and prepare the patient for minimal surgical intervention.

Regarding the etiological investigation, general surgery evaluation confirmed symmetrical pulses, indicating patent large arteries. The negative autoimmune tests and blood cultures ruled out secondary causes such as connective tissue diseases and septicemia. Nailfold capillaroscopy, although limited by edema and necrosis, did not show megacapillaries or microhemorrhages, typical of systemic sclerosis. The negative results from the computed tomography scan of the chest, abdomen, and pelvis ruled out underlying neoplasia and thrombosis, which are important considerations in the differential diagnosis. All these further support the diagnosis of severe secondary Raynaud's phenomenon, potentially triggered by COVID-19.

Primary Raynaud's phenomenon is generally a benign condition, and the risk of amputation is extremely low. The percentage of patients with severe Raynaud's phenomenon secondary to systemic sclerosis that requires amputation is around 4.8% [7]. This case report shows a rare case of severe Raynaud, probably secondary, that was refractory to both first and second-line therapies and required amputation.

## Conclusions

This case highlights the potential impact of COVID-19 as a precipitating factor for severe Raynaud's phenomenon, particularly in elderly patients with significant comorbidities. The rapid progression to necrosis despite standard and advanced treatments highlights the need for early recognition and aggressive management of Raynaud's phenomenon in similar high-risk patients. Further research is warranted to explore the potential pathophysiological links between COVID-19 and Raynaud's phenomenon and to develop more effective diagnostic tools and treatment strategies for refractory cases.
